# Site-specific plaque pH before and after short-term use of 1.5% Arginine dentifrice in caries-free and caries-active individuals

**DOI:** 10.1080/20002297.2017.1325255

**Published:** 2017-06-09

**Authors:** H Hassan, L Ghali, D Wildeboer, S Sarwar, P Lingström, A Carlén

**Affiliations:** ^a^ Dep. of Oral Microbiology and Immunology, Institute of Odontology, Sahlgrenska Academy, University of Gothenburg, Gothenburg, Sweden; ^b^ Department of Natural Science, School of Science & Technology, Middlesex University, London, UK; ^c^ Dep. of Cariology, Institute of Odontology, Sahlgrenska Academy, University of Gothenburg, Gothenburg, Sweden

## Abstract

**Objective**: To examine the pH response to an acidic challenge in site-specific plaque following a six-week period of using toothpaste consisting of 1.5% arginine and 1450 ppm fluoride or just using 1450 ppm fluoride toothpaste alone, in relation to caries.

**Methods**: Thirty-three healthy adults with no caries (caries-free) or ≥1 current manifest lesion (caries) participated in the study. Interproximal plaque-pH was measured in four sites. Two-weeks after professional cleaning, pH-measurements were performed before and up to 15-min after a 1-min rinse with 10 ml of a 10% sucrose solution. This procedure was repeated after a 6-week test period of using randomly selected toothpaste with 1.5% arginine and fluoride or with fluoride alone. The stimulated salivary flow rate was determined and the salivary-pH and buffer capacity was measured.

**Results**: In the caries group, the use of toothpaste with arginine and fluoride resulted in increased pH-values in the four sites tested. Although it was not statistically significant, increased pH-values were found in the caries-free group. In addition, the whole saliva buffer capacity and pH was increased in the caries group.

**Conclusion**: The most pronounced effect on plaque-pH and stimulated saliva after using arginine and fluoride toothpaste was seen in the caries group.

